# The Role of MicroRNAs in Selected Forms of Glomerulonephritis

**DOI:** 10.3390/ijms20205050

**Published:** 2019-10-11

**Authors:** Magdalena Nalewajska, Klaudia Gurazda, Ewa Styczyńska-Kowalska, Małgorzata Marchelek-Myśliwiec, Andrzej Pawlik, Violetta Dziedziejko

**Affiliations:** 1 Department of Nephrology, Transplantology and Internal Medicine, Pomeranian Medical University, Powstańców Wlkp 72 Str, 70-111 Szczecin, Poland; magda_nalewajska@yahoo.co.uk (M.N.); styczynskae@gmail.com (E.S.-K.); malgorzata.marchelek@gmail.com (M.M.-M.); 2 Department of Biochemistry and Medical Chemistry, Pomeranian Medical University, Powstańców Wlkp 72 Str, 70-111 Szczecin, Poland; gurazdaklaudia@gmail.com (K.G.); viola@pum.edu.pl (V.D.); 3 Department of Physiology, Pomeranian Medical University, Powstańców Wlkp 72 Str, 70-111 Szczecin, Poland

**Keywords:** microRNA, chronic kidney disease, IgA nephropathy, lupus nephritis, focal segmental glomerulonephritis, minimal chance disease

## Abstract

Glomerulonephritis (GN) represents a collection of kidney diseases characterized by inflammation within the renal glomeruli and small blood vessels. The lesions that occur in other nephron structures mainly result from the harmful effects of proteinuria. In recent years, an emphasis has been placed on gaining a better insight into the pathogenesis and pathophysiology of GN in order to facilitate diagnoses and provide efficient and targeted treatments of the disease. Owing to the advanced molecular and genetic diagnostic techniques available today, researchers have been able to elucidate that most cases of GN are determined by genetic risk factors and are associated with the abnormal functioning of the immune system (the immunologically mediated forms of GN). MicroRNAs (miRNAs) are a group of single-stranded, non-coding molecules, approximately 20 nucleotides in length, that act as regulatory factors in the post-transcriptional processes capable of regulating the expression of multiple genes. In this paper we present the available research aiming to determine effects of miRNAs on the development and progression of GN and discuss the potential role of miRNAs as new diagnostic markers and therapeutic targets.

## 1. Introduction

Glomerulonephritis (GN) is a non-homogeneous group of diseases characterized by inflammatory lesions within the renal glomeruli and small blood vessels. The lesions observed in other nephron structures are of a secondary nature and mainly result from the harmful effects of proteinuria. GN is caused by the improper functioning of immunological processes, although the detailed pathogenesis of most GN cases is yet to be understood. The large number of different forms of GN can be divided into two major subgroups: primary and secondary. Primary forms of GN are distinguished by pathological lesions that are initially only found in the glomeruli, where the clinical symptoms and laboratory test deviations are caused by disorders to their structure and function. Secondary forms of GN, however, originate from non-glomerular diseases that are rather systemic. Another way to classify GN is based on histopathological criteria distinguishing between proliferative and non-proliferative instances of GN. In most of the cases, histopathological lesions ensure the emergence of a typical set of clinical symptoms. Proliferative glomerulopathies are accompanied by the nephritic syndrome that is comprised of arterial hypertension, active urine sediment, oliguria, and proteinuria of <3.5 g/d, while non-proliferative glomerulopathies are accompanied by nephrotic syndrome, which is characterized by proteinuria >3.5 g/d, hypoalbuminemia, lipid disorders, and edemas [1].

In recent years, emphasis has been placed on gaining a better insight into the pathogenesis and pathophysiology of GN that could facilitate quicker diagnosis and a more efficient targeted treatment. Owing to the advanced molecular and genetic diagnostic techniques available today, researchers have discovered that most cases of GN are determined by genetic risk factors and the immunologically mediated forms of GN are associated with the abnormal functioning of the immune system. In genetically predisposed persons, environmental factors may cause overexpression of certain immunological factors which, when deposited in the glomeruli, lead to damage [2].

MicroRNAs (miRNAs) are a group of single-stranded, non-coding molecules containing approximately 20 nucleotides that act as regulatory factors of post-transcriptional processes. These small molecules regulate the expression of multiple genes. Up to 30% of the genes encoding human proteins are regulated by miRNAs [3,4]. miRNAs play a significant role in ensuring that normal functioning of the human body occurs by affecting, inter alia, cell migration, proliferation, differentiation, and apoptosis [4]. Incorrect miRNA expression may lead to the disturbance of important cellular processes, which, for example, can lead to oncogenesis [4]. Many papers published recently have pointed to the role played by miRNAs in the pathogenesis and development of chronic kidney disease, including various types of GN, among those mentioned were IgA nephropathy, focal segmental glomerulosclerosis (FSGS), and lupus nephritis (LN).

In this paper we review available reports on the effects of miRNAs on the development and progression of GN and discuss their new roles as diagnostic markers and potential therapeutic targets.

## 2. The Biogenesis and Regulatory Function of MicroRNAs

As mentioned above, miRNAs are single-stranded non-coding regulatory molecules responsible for modifying the expression of multiple genes, mainly in the post-transcriptional stage, although they have also shown to affect the process of transcription itself in certain cases. Most miRNAs are transcribed from DNA to pri- and then pre-miRNA, and finally cleaved to form mature miRNA. In a majority of cases, miRNAs interact with the three prime untranslated region (3’-UTR) of the target mRNA and cause translational inhibition or degradation of mRNA [5].

MicroRNAs were first identified in 1993 when Ambros and Ruvkun extracted the molecules from the model nematode, *Caenorhabditis elegans*. Researchers isolated the lin-4 molecule, responsible for the transition through larval stages [6,7]. Since then, more and more reports on a diverse array of miRNAs have been published, with the molecules being extracted from numerous animal models. Further, conserved, characteristic miRNA systems have been elucidated for many species. New miRNA molecules have continued to be discovered, further expanding knowledge of their biogenesis and role in gene regulation [5].

MicroRNAs form in several stages. A typical miRNA synthesis pathway begins in the nucleus, where the original transcript, or so-called primary miRNA (pri-miRNA), is transcribed. As a result of these hairpin-shaped structures processing, a precursor miRNA (pre-miRNA) is formed. The main functional role of this process is facilitated by the RNA-binding protein complex, DGCR8 (Di George syndrome critical region gene 8), and Drosha ribonuclease, an RNase III enzyme. DGCR8 binds to the single-stranded ends of the pri-miRNA and makes it possible for the RNase catalytic domain to specifically cleave transcripts to release pre-miRNA molecules approximately 60–100 nucleotides in length. The pre-miRNA is then transported by Exportin 5 to the cytoplasm, where as a result of the activity of the Dicer enzyme (another RNase III enzyme), it is cleaved into a double-stranded miRNA-miRNA* molecule (approx. 20 nucleotides in length). The miRNA-miRNA* complex is composed of the guide strand and the passenger strand; the latter being marked with an asterisk. By way of numerous transformations, the passenger strand is degraded and the guide strand is incorporated into the RISC complex containing argonaute (AGO) proteins, and as a mature single-stranded miRNA molecule, it is finally able to bind to the 3′ untranslated regions (3’-UTR) of mRNAs [4,5].

The Ago family proteins, mainly Ago2, are one of the most important components of the mRNA-induced silencing complex (mRISC). Ago proteins facilitate both mRNA degradation and translation silencing possible through facilitating interactions between microRNA molecules and their target mRNAs. Full complementarity of the mRNA:miRNA system results in mRNA separation that promotes transcript degradation, whereas partial complementarity results in translation inhibition [4].

miRNA molecules are of key importance to the functioning of the human system and are responsible for the normal course of many biological processes. Disturbance to miRNA expression promotes development of numerous diseases. For example, such a relationship has been observed with regard to chronic kidney disease. Further, miRNA molecules have been identified as components of extracellular fluids, including as components of urine and blood serum, and as a result they have the potential to be used as easily available, diagnostic biomarkers [3,5].

## 3. microRNAs in Chronic Kidney Disease

An association has been shown between individual miRNAs and their location in given organs. In 2004, miRNA molecules specific for the kidney were identified, namely miR-192, miR-194, miR-204, miR-215, and miR-216 [8]. In another study, the expression of miR-192 and miR-194 was shown to be higher in the renal cortex, and that of miR-27b in the medulla [9]. The pathology of different renal diseases is closely related to the exact location of lesions within the kidney. Renal diseases can be divided into two large groups: those associated with glomerular injury and those associated with damage to the tubulointerstitial structures. Extracting miRNAs related to the injury of specific renal structures will be very important in the context of identifying their roles as specific, diagnostic biomarkers for renal diseases.

### 3.1. microRNAs in IgA Nephropathy

IgA nephropathy (IgAN) is the most prevalent type of primary GN worldwide [10]. In IgAN patients, high serum concentrations of poorly galactosylated IgA1 can be found bound by autoantibodies to the IgA or IgG glycans. This leads to the formation of immune complexes that are subsequently deposited in the mesangium, and cause a number of lesions in renal tissue [11,12]. The clinical course of IgA nephropathy is usually mild, although in 40% of the cases it may lead to end-stage renal disease [13]. The diagnosis of IgAN is based on renal biopsy and a histopathological assessment of the biopsy specimen. Renal biopsy is an invasive procedure and, although safe in principle, may entail a certain level of risk to the patient. Presently, considerable attention is paid to finding non-invasive diagnostic markers for IgAN that would be easily available, quick, specific, and correlate with the formation of histopathological lesions and course of the disease. A relationship between miRNAs and the occurrence of IgAN has been implicated [14].

Numerous studies have shown that the expression of various miRNAs is altered in IgAN patients. A study published in 2018 identified 48 miRNAs from IgAN patients in which expression levels differed from healthy individuals of the control group [15]. Of those 48 molecules, four miRNAs having the highest-fold increases in expression in patients with IgAN relative to the control group were singled out; miR-148a-3p, miR-150-5p, miR-20a-5p, and miR-425-3p. The authors showed that simultaneous increases in the expression of these four miRNAs could predict IgAN disease with high sensitivity and specificity. Subsequently, researchers analyzed whether the presence of miRNAs depended on the degree of the histopathological lesions according to Lee’s classification. Results indicated that diagnostic sensitivity was higher in histopathological lesion stage I-II patients than III-IV stage patients. In the same study, correlations between serum concentrations of selected miRNAs and the clinical outcomes were also assessed. In these tests, researchers determined that the level of miR148a-3p correlated positively with eGFR, which could indicate the potential role of the miRNA as a marker that can predict eGFR changes in IgAN patients. In a study performed by Serino et al. two miRNA molecules, let7b and let148 were upregulated in peripheral blood mononuclear cells (PBMCs) [16]. A retrospective, multicenter study showed that joint determination of these two miRNAs allowed for distinguishing IgAN patients both from those suffering from other forms of GN (such as minimal change disease, membranous GN and FSGS) and healthy individuals [16,17].

In the study carried out by Wang et al. researchers showed that the expression of selected miRNAs in the urine of IgAN patients depended on the histopathological advancement of their lesions (Lee’s classification) as compared to patients with other GN forms, including minimal change disease and membranous GN [18]. Four miRNAs were uniquely expressed in the group of patients with stage I-II lesions and six were uniquely expressed in patients with stage III lesions. No changes were observed in miRNA expression levels in patients with stage IV and V lesions relative to the control group. This suggests that the changes in the levels of miRNA expression of IgAN patients may be dynamic and related to the progression of the disease and its level of severity [18]. In another study, an association between urine miRNA content and the clinical stage of the disease was shown in which a negative correlation was observed between the levels of miR-200b in the urine of IgAN patients and the dynamics of changes related to rates of glomerular filtration (eGFR). In this case, lower levels of miRNA-200b levels were associated with faster drops in eGFR values [19]. Recently published study by Szeto et al. verified the usability of quantified urinary miRNAs in patients with IgA nephropathy [20]. The levels of urinary miR-204, miR-431, and miR-555 were significantly reduced, but miR-150 significantly increased in the IgA group compared to healthy controls. It was shown that among the approved miRNA, miR-204 has the best diagnostic accuracy, however, all of the other targeted miRNAs (miR-150, miR-431, miR-555) also exhibited the diagnostic accuracy. Furthermore, the urinary levels of miR-555 were correlated with the duration of disease and baseline estimated GFR. Unfortunately, none of the studied miRNAs has revealed correlation with the estimated GFR decline or predicted disease progression.

An increasing number of studies have pointed to miRNAs having roles in the pathogenesis of IgA nephropathy [17]. The significance of miR148b, 374b, and let-7b in regulating the galactosylation of IgA1 in PBMCs has been shown. miR148b and let-7b inhibit the expression of the C1GALT1 and GALNT2 enzymes at the mRNA and proteins expression levels, whereas miR148b correlated positively with the concentration of poorly galactosylated IgA1 [21]. Another study showed that miR374b was overexpressed in B cells within the Chinese population and that the molecule had an inhibitory effect on selected chaperone proteins and suppressor genes, resulting in increased B-cell proliferation and higher serum concentrations of poorly galactosylated IgA1 [22].

miRNAs are also believed to play an essential role in the pathogenesis of IgAN by regulating the release of proinflammatory cytokines [3]. It has been shown that miR16, 100-3p, and 877-3p negatively regulate IL-6, IL-8, and IL-1, respectively, through affecting translation [23,24]. Expression of miR-29b-3p is diminished in patients with IgAN. miR-29b-3p negatively regulates CDK6 kinase and inhibition of the expression of this miRNA increases p65 phosphorylation and induces the TNF-α-dependent expression of IL-8 [25]. Another study showed that miR21 expression was increased in the renal tissue of IgAN patients. Inhibition of miR21 expression reduced the expression of fibrosis markers and PTEN activation, which suggested that miR21 may play a role in the progression of renal tissue fibrosis in patients with IgAN [26].

Perhaps in the future, apart from their role in the diagnosis of renal diseases, miRNAs will become therapeutic targets for IgAN treatment. Inhibiting the activity of selected miRNAs could lead to blocking the first stage of pathogenesis of IgAN, thus reducing the levels of poorly galactosylated IgA1, or curbing renal tissue fibrosis, thus slowing down the progression of disease. Nonetheless, it should be remembered that each of the miRNAs is responsible for multiple transcriptional processes, which is why miRNA inhibitors need to be used cautiously in order to minimize the risk of inducing adverse effects [17].

### 3.2. microRNAs in Lupus Nephritis

Systemic lupus erythematosus (SLE) is an autoimmune disease of unknown etiology, which is characterized by inflammation that can be found in a large set of tissues and organs. One of the most frequent organ manifestations of SLE is LN, which in many cases leads to end-stage renal disease. In the progression of LN, immune complexes are deposited or formed in situ in the kidney, which invokes cytokine responses and leads to damage to the endothelium of the glomerular capillary loops and small renal vessels, podocytes, tubules, and renal interstitium. Lesions in the kidneys are observed in approximately 50% of patients diagnosed with SLE. The degree of renal lesions is assessed on the basis of biopsies and the histopathological classification of the lesions is done according to ISN/RPS (International Society of Nephrology/Renal Pathology Society), which distinguishes VI classes [1].

The first association between miRNAs and LN was shown in 2009 by Dai et al. who identified 66 miRNAs in renal biopsy specimens from LN patients in which the expression of miRNAs was modified as compared to healthy individuals of the control group [27]. In another study, five miRNAs were singled out in PMBCs, namely hsa-miR-371-5P, hsa-miR-423-5P, hsa-miR-638, hsa-miR-1224-3P, and hsa-miR-663, in which expression levels were modified in LN patients independent of race [28]. Reduced levels of miR-200a, miR-200c, miR-141, miR-429, and miR-192 have also been observed in LN patients in comparison to healthy individuals [29]. Increased miRNA-146a content in the renal tissue affected by LN lesions has also been shown, while no elevated expression of the same molecule was found in the healthy renal parenchyma [30]. The miRNA composition of urine sediment of LN patients may facilitate diagnosis, since it has been shown that the determination of urine miR-192 and miR-27b levels can be used as a diagnostic marker in patients with LN [31].

Recently, studies have also confirmed the important role of miRNAs in LN. In a 2018 study, elevated plasma concentrations of miR-125a, miR146a, and miR155 were shown in LN patients compared to the control group comprising healthy individuals [32]. The plasma content of miR146a has also been correlated with the anti-dsDNA levels and proteinuria, whereas the levels of miR-142-3p correlate with the clinical course of the disease. Moreover, a joint determination of the plasma content of miR-125a, miR-142-3p, miR-146, and miR-155 provided a high-sensitivity and high-specificity means for diagnosing LN. In another study, miR-146a-5p displayed a positive correlation with creatinine content, but not with the levels of albumin and the urine protein to creatinine ratio. The authors suggested that miR-146a-5p could be used as a marker for early stages of the disease, where the plasma content of albumin and the urine protein to creatinine ratio are still within the norm [33]. In a study published in 2019, the PBMC miRNA concentration was determined in women with SLE, where kidneys were not affected, those with inactive disease, in patients with active LN, and a group of healthy individuals [34]. An increased expression of miR-21 and miR-155 was found in patients with active LN when compared to all of the other groups. Moreover, the authors suggested that miR-21 and miR-155 could potentially serve as potential diagnostic markers of LN.

In a study by Navarro-Quiroz et al. not only differences in abundance of plasma circulating microRNAs in LN have been measured, but also particular microRNAs have been assessed as potential contributors to LN pathogenesis [35]. In this study, increased levels of plasma miR-107-3p have been observed in LN individuals compared to healthy subjects. The authors conclude that altered miR-107-3p concentrations contribute to LN manifestation by causing a deficiency in E-cadherin and therefore causing disturbed intercellular communication. Lower levels of another microRNA – miR-375-3p observed in LN patients are believed to cause overexpression of SLK (Ste-20 like-kinase) leading to dysregulation of podocyte cytoskeleton and as a consequence to proteinuria. Likewise, miR-150 overexpressed in LN induces synthesis and release of profibrotic molecules through JAK/STAT signaling pathway contributing to cell-proliferation, inflammatory state, and fibrosis [36].

### 3.3. microRNAs in Focal Segmental Glomerulosclerosis and Minimal Change Disease

FSGS, or focal segmental glomerulosclerosis, is comprised of group of forms of GN that are caused by podocyte injury resulting in subsequent glomerulosclerosis and mesangial matrix expansion [37]. FSGS is a nephrotic syndrome that occurs both in adults and children [14]. Currently, the diagnosis of FSGS is solely based on a histopathological assessment of renal biopsy specimens, and no non-invasive diagnostic markers for this type of GN have been yet identified. Minimal change disease (MCD), is another nephropathy capable of developing into nephrotic syndrome. It is characterized by a lack of visible abnormalities when histopathological sections of renal biopsy specimens are observed using a light microscope; however, when viewed with an electron microscope, characteristic lesions in the form of flattened podocyte foot processes are visible [38]. Although MCD diagnosis is quite simple in children since the disorder is responsible for most nephrotic syndrome cases of this age group. Therefore, in children, the diagnosis can be made based on clinical symptoms and the response to steroid therapy without taking renal biopsies. For adults, however, diagnosing MCD can be more problematic. Therefore, finding markers specific for this disease, e.g., by identifying specific marker microRNAs, could expand diagnostic possibilities with respect to MCD considerably and facilitate the monitoring of the course of the disease in patients.

In 2013, a study was published which assessed the association of miR-192 and miR-205 with the occurrence and course of FSGS and MCD [39]. The FSGS patients had higher plasma concentrations of both miRNAs tested than MCD patients. miR-192 content correlated positively with the severity of proteinuria both in the FSGS and MCD groups of patients, whereas the content of mi-205 correlated positively with proteinuria only in the FSGS group. Further, the miR-192 concentration correlated with the degree of interstitial fibrosis in FSGS patients. The authors drew the conclusion that the tested miRNA molecules could be used as a marker differentiating these two diseases. In a 2015 pilot study, miRNA concentrations in FSGS and MCD patients were also assessed [40]. In this study, 126 and 155 miRNAs were extracted from plasma and urine, respectively, the concentrations of which differed between FSGS and MCD patients. The plasma levels of miR-30b, miR-30c, miR-34b, miR34c, miR342, and miR-1225-5p were higher in MCD patients than both FSGS patients and the control group of healthy individuals, while the urine levels of miR-1915 and miR-663 were lower in FSGS patients than in patients with MCD, and the urine content of miR-155 in patents with FSGS was higher than those suffering from MCD and the control group. In their initial conclusions, the authors assumed that the miRNA profile was characteristic of each of these disease entities, and further research could contribute to identifying specific markers useful in differentiating both diseases. Another study assessed the association between urine concentrations of selected miRNAs and the activity and clinical course of FSGS [41]. miR-196a, miR-30a-5P, and miR-490 differentiated the active from of the disease from FSGS in remission. After steroid therapy, concentrations of the miRNAs dropped in those patients who demonstrated a response to the treatment and remained stable in those without any clinical response. Through this analysis, a relationship was demonstrated between the levels of these miRNAs and FSGS activity. In a more recent 2018 study, researchers found that the plasma levels of miR-17, miR-451, miR-106a, and miR-19b were significantly reduced in FSGS patients compared to the healthy individuals [14]. In the same study, the panel of the studied miRNAs correlated with histopathological lesions secondary to FSGS, but no such correlation was found for clinical parameters, such as glomerular filtration rate, the degree of proteinuria, or the plasma concentration of albumin. The upregulated expression of three of the studied miRNAs, miR-17, miR-451, and miR-106a, was associated with complete remission of FSGS.

The microRNAs play also an important role in the pathogenesis of FSGS [36]. As stated above, FSGS is caused by the occurrence of podocyte injury. The group of mRNAs – miR-30, miR-132, miR-134, and miR-29a – is highly expressed in human podocytes, showing a beneficial effect in protection against apoptosis and in controlling the calcium/calcineurin signaling pathway. This pathway is responsible for valid maintenance of podocyte cytoskeleton and its disturbed functioning can lead to FSGS manifestation. Therefore, the expression levels of miR-30 and its family are lower in patients with FSGS. Moreover, increased levels of miR-193a have been identified in individuals with FSGS in comparison to healthy kidneys and other kidney diseases [36]. The over expression of this microRNA has been linked to an abnormal activation of podocytes and formation of extracapillary lesions observed in FSGS. Following the evidence that the occurrence of crescents within glomeruli in FSGS patients could be reversed after targeting the miR-193a, there is a hope for miR-193a to become a therapeutic target for FSGS treatment.

## 4. Conclusions

Despite the advancements in medicine, the diagnosis and monitoring of the disease course and response to treatment of different forms of GN remain a challenge to clinicians. Currently, a histopathological assessment of renal biopsy specimens is the only commonly practiced diagnostic mean to evaluate GN. Renal biopsy is an invasive method, which is why it is important for new, non-invasive diagnostic markers to identify GN. Presently, microRNAs, single-stranded non-coding nucleotide molecules responsible for regulating the expression of multiple genes, are attractive potential markers of GN. Some evidence also suggests the role of miRNA in the pathogenesis or as potential therapeutic agents, especially in IgA nephropathy. Nonetheless, further multicenter studies are required in order to assess the exact utility of microRNAs in the general diagnosis and monitoring of the course of selected forms of GN. The exact role of microRNAs as therapeutic strategies in GN is also yet to be determined.
